# Multidisciplinary management of extensive cervical lymphatic malformations and epiglottic cyst in a neonate: a case report and review of perioperative strategies

**DOI:** 10.3389/fped.2025.1543250

**Published:** 2025-09-08

**Authors:** Yuzhu Cai, Lingli Zhang, Jun Wang, Yingying Sun

**Affiliations:** Department of Anesthesiology, Anhui Provincial Children’s Hospital, Hefei, China

**Keywords:** lymphatic malformations, epiglottic cyst, neonatal airway obstruction, laser ablation, sclerotherapy, general anesthesia

## Abstract

Lymphatic malformations (LMs) are rare congenital anomalies that can cause life-threatening airway obstruction in neonates, particularly when located in the cervicofacial region. The coexistence of lymphatic malformations with an epiglottic cyst further exacerbates airway compromise, posing significant diagnostic and therapeutic challenges. We report the case of a 27-day-old neonate with extensive cervical lymphatic malformations and an epiglottic cyst causing severe airway obstruction. Timely intervention, including laser ablation of the epiglottic cyst and sclerotherapy for the lymphatic malformations, was successfully performed under general anesthesia. Perioperative management emphasized safe airway control and meticulous surgical planning. Postoperative outcomes were favorable, with significant reduction in lymphatic malformations size and resolution of airway obstruction after additional sclerotherapy sessions. This case underscores the importance of a multidisciplinary approach, combining advanced imaging, innovative airway management techniques, and minimally invasive procedures, to achieve optimal outcomes in complex neonatal airway and vascular anomalies.

## Introduction

Lymphatic malformations (LMs) are rare congenital vascular anomalies arising from abnormal lymphatic system development (1). They most commonly affect the cervicofacial region (2), varying in size and complexity, and are often characterized by large cystic masses. In neonates, extensive LMs can cause significant complications by compressing adjacent structures, particularly in the head and neck region, potentially leading to airway obstruction, feeding difficulties, and other life-threatening conditions (3). The coexistence of LMs with epiglottic cysts presents unique diagnostic and therapeutic challenges due to their critical impact on airway management.

Epiglottic cysts is a rare but potentially lethal supraglottic airway pathology in infants and are caused by ductal obstruction of either mucous glands or minor salivary glands in the vallecula and base of the tongue (4).While congenital epiglottic cysts are non-malignant growths, in neonates and infants, substantial cysts located in the epiglottis can potentially obstruct the airway, leading to either respiratory impediments or instances of food inhalation that might result in foreign body obstruction of the trachea. Such occurrences elevate the potential peril of death by asphyxia (5). Their presence exacerbates airway compression symptoms caused by extensive cervical LMs, necessitating prompt evaluation and intervention. Advances in imaging techniques, such as computed tomography (CT), play a critical role in diagnosing and characterizing these complex lesions, while airway endoscopy provides vital insights into the extent and nature of airway involvement. Multidisciplinary collaboration is essential for devising personalized and comprehensive management strategies.

Management of neonatal LMs and associated epiglottic cysts often involves a combination of surgical and minimally invasive interventions. Laser ablation of epiglottic cysts provides rapid relief of airway obstruction (6), whereas sclerotherapy offers a minimally invasive alternative for reducing LMs size (7). Optimal perioperative airway management is crucial, as neonates with compromised airways are at high risk of complications during anesthesia induction and intubation.

This report details a rare case of a 27-day-old neonate with extensive cervical LMs and an associated epiglottic cyst causing severe airway obstruction. The patient underwent successful perioperative management under general anesthesia, including laser ablation of the epiglottic cyst and localized sclerotherapy for the LMs.

## Case report

**Figure 1:**
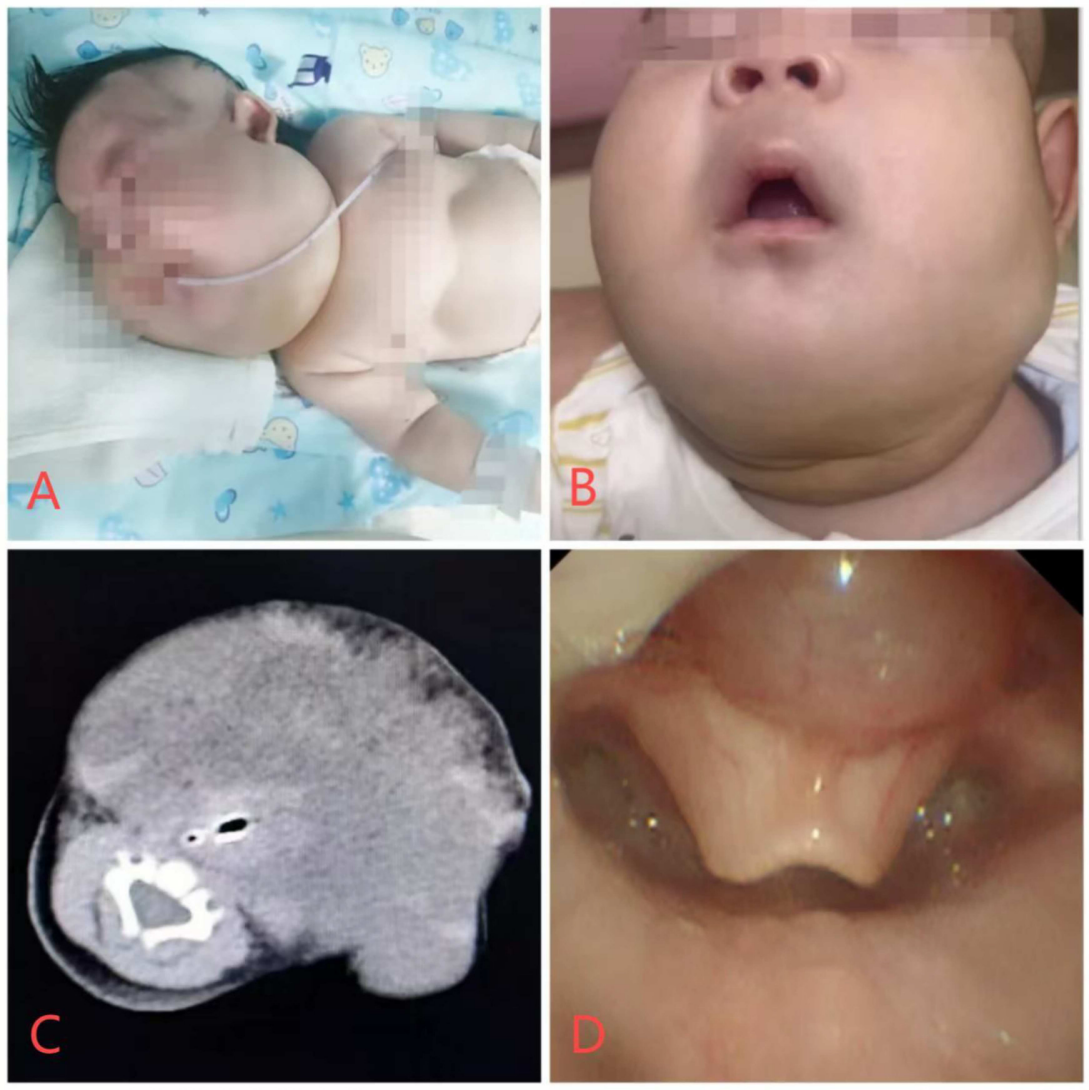
Patient photographs and imaging data. **(A)** Presence of suprasternal, supraclavicular, and intercostal retractions upon admission. **(B)** The patient's 8-month-old cervical lymphatic malformation has significantly reduced in size. **(C)** Cervical and Head CT Image of the Patient. **(D)** Bronchoscopic view of an epiglottic cyst.

The patient, a 27-day-old female neonate, measured 52 cm in length and weighed 4.15 kg at the time of admission. Prenatal monitoring revealed a multilocular cystic mass in the neck and submandibular region. At 27 days postpartum, the patient presented with respiratory distress and positive signs of retractions (Figure 1A). Born at 39 weeks + 1 day gestation with a birth weight of 3.75 kg, she was admitted for further evaluation. Treatment included nasal oxygen, thermal support, vitamin K1 supplementation, monitoring of blood pressure and glucose, anti-infective therapy, formula feeding, and intravenous nutritional support.

The computed tomography (CT) scan of the head and neck was performed to delineate the extent and nature of the lymphatic malformations (LMs) and the epiglottic cyst in the neonate. The scan revealed extensive hypodense areas in the bilateral maxillofacial and submandibular regions, with scattered hyperdense signals, indicative of hemorrhagic LMs. The lesions measured approximately 62 mm × 45 mm × 44 mm. (Figure 1C). The presence of large hypodense areas suggested the presence of cystic structures typical of LMs. The scattered hyperdense signals within these areas were consistent with hemorrhagic changes. Bronchoscopy revealed an epiglottic cyst and mild tracheomalacia in the upper trachea (Figure 1D). A distinct cystic structure was identified at the base of the epiglottis. This cyst was visualized using bronchoscopy, confirming its location and the degree of airway obstruction it caused. The CT scan provided crucial information for the multidisciplinary team (MDT) to plan the surgical intervention. The extent and location of the LMs and the epiglottic cyst were meticulously assessed to determine the most appropriate approach for laser ablation and sclerotherapy. The MDT decided on an emergency laser ablation of the epiglottic cyst to alleviate immediate airway obstruction, followed by intralesional sclerotherapy for the LMs to reduce their size and prevent future complications.

Following peripheral venous access in the ward, the patient was transferred to the operating room at 14:30, with baseline monitoring showing SpO_2_ of 94%, HR of 135 bpm, and BP of 82/49 mmHg. The neonate was positioned laterally with a 30° elevation to optimize respiratory function. Preoxygenation with 6 L/min oxygen was administered via face mask for 5 min. Before induction, the airway cart was checked, including rescue equipment such as supraglottic airway devices (I-Gel), with an otolaryngologist on standby for emergency tracheotomy.

Anesthesia was induced using sevoflurane, and intubation was performed using a 3.5 mm uncuffed endotracheal tube and a video laryngoscope (Insight iS2). The epiglottic cyst was visualized and lifted with the laryngoscope for clear exposure of the glottis. Intubation was successful without complications. Cisatracurium (0.5 mg) was administered for muscle relaxation, and anesthesia was maintained with 1.5% sevoflurane and continuous remifentanil infusion (0.2–0.25 µg/kg/min). Dexamethasone (1 mg) was administered prophylactically to prevent postoperative airway edema.

The oxygen concentration was reduced to 40% during surgery to minimize fire risk during laser ablation of the cyst. However, to ensure patient safety with this reduced oxygen level, arterial blood gas analysis was performed every 10 min, and the oxygenation index was continuously monitored and adjusted according to the patient's condition to ensure adequate tissue oxygenation. Laser ablation was performed using a CO_2_ laser (UltraPulse®, Lumenis, Santa Clara, CA, USA) with a power of 10 W and a spot size of 1 mm. The procedure was performed transorally under bronchoscopic guidance. The procedure lasted 20 min, followed by 26 min of sclerotherapy under interventional guidance. for the LMs. Sclerotherapy was performed using Bleomycin at a dose of 0.5 mg/kg, injected directly into the cystic spaces of the lymphatic malformations. The neonate was transferred intubated to the PICU postoperatively and successfully extubated 24 h later. She was discharged on postoperative day five in stable condition.

The patient underwent three additional sclerotherapy treatments under general anesthesia at 3 months, 5 months, and 8 months of age, with significant reduction in LMs size and favorable outcomes (Figure 1B).

## Discussion

This case highlights the successful management of a rare combination of extensive cervical LMs and epiglottic cyst in a neonate. The severe airway obstruction posed a significant challenge, requiring a multidisciplinary approach emphasizing meticulous preoperative planning, timely surgical intervention, and careful perioperative management.

Imaging modalities, particularly CT, were instrumental in delineating the extent of the lesions and guiding interventions. The use of a video laryngoscope facilitated safe airway management and direct visualization of the epiglottic cyst, guiding laser ablation (8). Laser ablation, as a minimally invasive technique, effectively relieved airway obstruction while minimizing collateral tissue damage. Special precautions were taken to reduce intraoperative oxygen concentrations, as high oxygen levels increase the risk of airway fire.Lasing in more than 50% oxygen is comparatively dangerous and can cause airway fire in less than 5 s (9). For every 10% increase in oxygen concentration above 60% the risk of flame increased by a factor of 2.3 (10).

Sclerotherapy provided a valuable minimally invasive option for LMs management, significantly reducing lesion size without the morbidity associated with open surgical excision (11). Perioperative measures, including preoxygenation, equipment readiness, and dexamethasone administration, minimized the risk of catastrophic airway complications and postoperative edema.

In this case, we did not perform drainage or place a drainage tube for the lymphatic malformations. The MDT concluded that the primary cause of airway obstruction was the epiglottic cyst, rather than the cervical lymphatic malformations. Ultrasonography revealed that the lymphatic malformations were predominantly multicystic in nature. Placing a drainage tube might not have resolved the issue and could have potentially led to intracystic hemorrhage, worsening airway obstruction. However, if the cervical lymphatic malformations had posed a direct threat to airway safety, primary drainage would have been the preferred option.

The patient's favorable outcomes underscore the importance of MDT collaboration involving anesthesiologists, pediatric surgeons, otolaryngologists, and interventional radiologists (12). Long-term follow-up is essential, as ongoing sclerotherapy treatments may be required for sustained lesion reduction.

## Conclusion

This case highlights the importance of a multidisciplinary approach in managing rare neonatal conditions such as extensive cervical LMs coexisting with an epiglottic cyst, which posed a significant airway obstruction risk. The combination of timely surgical interventions, including laser ablation of the epiglottic cyst and sclerotherapy for the LMs, along with meticulous perioperative airway management, ensured a favorable outcome. This report underscores the critical role of careful preoperative planning, innovative airway management techniques, and long-term follow-up in achieving successful treatment outcomes for complex neonatal airway and vascular anomalies.

## Data Availability

The original contributions presented in the study are included in the article/Supplementary Material, further inquiries can be directed to the corresponding author.

## References

[1] Bouwman FCM Klein WM de Blaauw I Woiski MD Verhoeven BH Botden SMBI. Lymphatic malformations adjacent to the airway in neonates: risk factors for outcome. J Pediatr Surg. (2021) 56(10):1764–70. 10.1016/j.jpedsurg.2021.03.011 33902896

[2] Kronfli AP McLaughlin CJ Moroco AE Grant CN. Lymphatic malformations: a 20-year single institution experience. Pediatr Surg Int. (2021) 37(6):783–90. 10.1007/s00383-021-04859-5 33586010

[3] Honnorat M Viremouneix L Ayari S Guibaud L Coste K Claris O Early adjuvant medication with the mTOR inhibitor sirolimus in a preterm neonate with compressive cystic lymphatic malformation. Front Pediatr. (2020) 8:418. 10.3389/fped.2020.00418 32850534 PMC7399047

[4] Mulcahy CF Reddy SK Wikner EE Mudd PA. Neonatal airway anomaly: vallecular cyst. BMJ Case Rep. (2017) 2017:bcr2017223082. 10.1136/bcr-2017-223082 29141934 PMC5695396

[5] Guo S Cao C Feng C Li Z Zhou F Ye H Case reports of low-temperature plasma radiofrequency treatment for congenital epiglottic cysts in neonates and infants. Ear Nose Throat J. (2023). 10.1177/01455613231199699 37800471

[6] Chen D Duan M. Clinical effect of CO2 laser resection of the epiglottic cyst under micro-laryngoscope suspension. Acta Otolaryngol. (2022) 142(5):443–7. 10.1080/00016489.2022.2079717 35654408

[7] Bouwman FCM Kooijman SS Verhoeven BH Schultze Kool LJ van der Vleuten CJM Botden SMBI Lymphatic malformations in children: treatment outcomes of sclerotherapy in a large cohort. Eur J Pediatr. (2021) 180(3):959–66. 10.1007/s00431-020-03811-4 33051716 PMC7886713

[8] Meng X Wen Q Gu J Wang Y. Videolaryngoscope-assisted coblation of epiglottic cysts. Eur Arch Otorhinolaryngol. (2020) 277(4):1129–32. 10.1007/s00405-020-05804-3 31993766

[9] Stuermer KJ Ayachi S Gostian AO Beutner D Hüttenbrink KB. Hazard of CO₂ laser-induced airway fire in laryngeal surgery: experimental data of contributing factors. Eur Arch Otorhinolaryngol. (2013) 270(10):2701–7. 10.1007/s00405-013-2521-1 23636479

[10] Huang L Badenoch A Vermeulen M Ullah S Woods C Athanasiadis T Risk of airway fire with the use of KTP laser and high flow humidified oxygen delivery in a laryngeal surgery model. Sci Rep. (2022) 12(1):543. 10.1038/s41598-021-04636-3 35017619 PMC8752812

[11] Hu F Ma F Liu X Yu J. Sclerothrapy of giant lymphatic malformation in neonates. J Perinatol. (2025) 45(2):213–7. 10.1038/s41372-024-02113-z 39313546 PMC11825356

[12] Scuglia M Conforti A Valfrè L Totonelli G Iacusso C Iacobelli BD Operative management of neonatal lymphatic malformations: lesson learned from 57 consecutive cases. Front Pediatr. (2021) 9:709223. 10.3389/fped.2021.709223 34490164 PMC8416514

